# Food insecurity in adults with mood disorders: prevalence estimates and associations with nutritional and psychological health

**DOI:** 10.1186/s12991-015-0059-x

**Published:** 2015-07-17

**Authors:** Karen M Davison, Bonnie J Kaplan

**Affiliations:** Department of Community Health Sciences, University of Calgary, Calgary, AB Canada; Department of Biology, Health Science Program, Kwantlen Polytechnic University, 12666 72nd Avenue, Surrey, BC V3W 2M8 Canada; Department of Pediatrics, University of Calgary, Calgary, AB Canada; The Alberta Children’s Hospital Research Institute, Calgary, AB Canada

**Keywords:** Food insecurity, Nutrient intakes, Mood disorders

## Abstract

**Background:**

Because little is known about food insecurity in people with mental health conditions, we investigated relationships among food insecurity, nutrient intakes, and psychological functioning in adults with mood disorders.

**Methods:**

Data from a study of adults randomly selected from the membership list of the Mood Disorder Association of British Columbia (*n* = 97), Canada, were analyzed. Food insecurity status was based on validated screening questions asking if in the past 12 months did the participant, due to a lack of money, worry about or not have enough food to eat. Nutrient intakes were derived from 3-day food records and compared to the *Dietary Reference Intakes (DRIs)*. Psychological functioning measures included Global Assessment of Functioning, Hamilton Depression scale, and Young Mania Rating Scale. Using binomial tests of two proportions, Mann–Whitney *U* tests, and Poisson regression we examined: (1) food insecurity prevalence between the study respondents and a general population sample from the British Columbia Nutrition Survey (BCNS; *n* = 1,823); (2) differences in nutrient intakes based on food insecurity status; and (3) associations of food insecurity and psychological functioning using bivariate and Poisson regression statistics.

**Results:**

In comparison to the general population (BCNS), food insecurity was significantly more prevalent in the adults with mood disorders (7.3% in BCNS vs 36.1%; *p* < 0.001). Respondents who were food-insecure had lower median intakes of carbohydrates and vitamin C (*p* < 0.05). In addition, a higher proportion of those reporting food insecurity had protein, folate, and zinc intakes below the *DRI* benchmark of potential inadequacy (*p* < 0.05). There was significant association between food insecurity and mania symptoms (adjusted prevalence ratio = 2.37, 95% CI 1.49–3.75, *p* < 0.05).

**Conclusions:**

Food insecurity is associated with both nutritional and psychological health in adults with mood disorders. Investigation of interventions aimed at food security and income can help establish its role in enhancing mental health.

## Background

Among health researchers, policy makers, practitioners, and decision makers, there are concerns about the growing global and ethical issue of food insecurity [1], defined as the limited or uncertain availability of nutritionally adequate and safe foods or limited or uncertain ability to acquire acceptable food in socially acceptable ways [2]. The impact of food insecurity on mental health is significant and may be attributed to its associations with suboptimal diet [3–5], and psychological issues such as depression, eating disorders, and impaired cognition [6–9]. Of the few investigations that have examined food insecurity in populations with confirmed diagnosis of a mental health condition, results have indicated an association with food insufficiency [10] and that patients in a psychiatric emergency unit who lacked food security had higher levels of psychological distress compared to food-secure individuals [11]. In a previous study of the determinants of food intakes in a sample of adults with confirmed mood disorders conducted by the authors [12], a high prevalence of inadequate intakes of several micronutrients (e.g., folate, vitamin B_12_, iron, zinc) was found. As part of this investigation, the authors also included food insecurity screening questions and measures of psychological symptoms, as it was speculated that food access would be associated with both dietary intake and mental function in this population. To further understanding of food insecurity in specific mental health populations, we used data from the study of adults with mood disorders to answer the following research questions: (1) what is the prevalence of food insecurity compared to a general population sample?; and (2) is food insecurity associated with poorer nutrient intakes and psychological function? It was hypothesized that in this sample of adults with mood disorders, food insecurity would be: (1) significantly more prevalent than in the general population; (2) associated with suboptimal nutrient intakes based on defined standards; and (3) associated with poorer psychological functioning.

## Methods

### Sample

The data were from a cross-sectional nutrition survey of adults (>18 years) with clinically defined mood disorders (*Structured Clinical Interview for DSM*-*IV*-*TR Axis I Disorders* or SCID; [13]) who lived in the lower mainland of British Columbia; 146 individuals were randomly selected from the membership list of the Mood Disorder Association of British Columbia and invited to participate. Of the 146 randomly selected members, 26 were deemed ineligible and 23 refused to participate. Eleven of the 23 individuals who declined participation completed a non-response questionnaire, and statistical comparisons showed no significant differences compared to the respondents based on a variety of lifestyle habits (e.g., smoking, multivitamin use).

As part of the recruitment process, all individuals received phone follow-up from research staff to determine their interest and eligibility. Full details of recruitment, data collection, quality control, and overall nutrient intake results have been published previously [12]. This study adhered to guidelines in the Declaration of Helsinki and was approved by the University of Calgary’s Conjoint Health Research Ethics Board. Sufficiency of statistical power (minimum 80%) to conduct the analysis was based on: (1) the hypothesis, food insecurity would be associated with lower nutrient intakes; (2) results from a Canadian study on food insecurity and nutrient intakes reporting mean intakes of folate of 424 and 378 micrograms for food-secure and food-insecure groups, respectively, with a common standard deviation of 18.5 [14]; and (3) an alpha level of 0.05 [15].

### Measurements

#### Dependent variable

##### Food insecurity

Food insecurity status was determined as having an affirmative response to at least one of the two screening questions:

1. In the past 12 months did you worry that there would not be enough to eat because of lack of money? (Question description: Worry about food access).
2. In the past 12 months did you not have enough food to eat because of lack of money? (Question description: Compromised diet).

These two screening questions, which have previously been used in Statistics Canada national surveys [16], were also used in the British Columbia Nutrition Survey and allowed for direct comparisons of food insecurity between the sample and the general population. In the Canadian survey and in our study, a third question was also used: “In the past 12 months, did you or anyone in your household not have enough food to eat because of a lack of money?”. All of our study respondents who responded affirmatively to question two also indicated “yes” to the third question. Due to this redundancy in responses, the third question was not included in the analysis.

#### Independent variables

##### Psychological functioning and symptoms

Psychological, social, and occupational functioning was based on scores of the Global Assessment of Functioning Scale (GAF) [17]. Current symptoms of depression and mania were measured based on the Hamilton Depression Scale (Ham-D) [18], and Young Mania Rating Scale (YMRS) [19].

##### Diet

Nutrient intakes were derived from 3-day food records (two weekdays and one weekend day; non-consecutive) according to standard protocols [20]. The dietary variables included intakes of energy, macronutrients, and selected micronutrients (i.e., vitamins B_6_, B_9_, B_12_, and C, plus the trace minerals iron, and zinc) that function in cognition, and were analyzed using Food Processor SQL with the Canadian nutrient file [21].

#### Covariates

Covariates included in the analysis were body mass index (BMI; kg/m^2^; continuous variable) measured according to standard protocols used in national and provincial nutrition surveys [20], diagnosis of depressive or bipolar disorder based on the SCID (dichotomous), following a therapeutic (diet for a medical condition) diet (dichotomous), sociodemographic factors including age (continuous), gender (male/female), in a relationship or not (dichotomous), and income (dichotomized as low versus adequate, based on Canadian government standards).

### Data analysis

To test the first hypothesis, prevalence estimates of food insecurity (i.e., affirmative response to at least one of the two food insecurity screening questions) and low income were compared (using binomial tests of two proportions) to regional data available from the 1999 British Columbia Nutrition Survey (BCNS; *n* = 1,823) that contained the same income and food insecurity measures [20]. The BCNS was conducted by the British Columbia Ministry of Health Services, Health Canada, and the University of British Columbia. It randomly sampled British Columbians aged from 19 to 84 years. Most of the BCNS participants were married (65.2%), did not hold a university degree (85.5%), and had income levels above the poverty cut-off (74.8%).

To examine the second hypothesis, differences in nutrient intakes between respondents who were either food-insecure or secure were compared using Mann–Whitney *U* tests or binomial tests of two proportions based on the *Dietary Reference Intakes* [22]. Specifically, the proportion of the sample above and below the Estimated Average Requirements (EAR; to estimate prevalence of possible nutrient inadequacy) or within and above the Adequate Macronutrient Distribution Ranges (AMDR; to estimate suboptimal or excess macronutrient intake) were analyzed.

To test the third hypothesis, bivariate relationships between food insecurity and the psychological measures were analyzed using the Mann–Whitney *U* test and crude prevalence ratios. Cut-offs used to categorize the mental health measures included scores of 7 or less for the Ham-D [18] and 12 or less for the YMRS [19] which indicate that symptoms are currently absent. A cut-off of 60 for the GAF was used; scores less than this indicate more severe mental illness [23].

The final steps of the analysis involved conducting Poisson regression with robust variance to examine relationships between food insecurity and psychological functioning while adjusting for age, sex, relationship status, and income. This particular method was chosen as it is considered the most viable model option when analyzing cross-sectional data [24]. It provides adjusted prevalence ratios that are interpreted as the ratio of the proportion of the persons with the outcome (e.g., low GAF scores) over the proportion with the exposure (e.g., food insecurity). To test whether the Poisson model form fit the data, the goodness-of-fit Chi-squared test was applied. All data analyses were performed using Stata 10.0 [25].

## Results

### Description of sample

Similar to the general population sample studied in the BCNS, most respondents (*n* = 97) were females (*n* = 69; 71.1%). Unlike the BCNS, most did not have a university degree (*n* = 76; 78.4%) and were not in a relationship (61.9%). Approximately half of the sample (49.0%) had government defined low-income levels (Statistics Canada, 2013). Most (67.0%) had body mass indices greater than 24.9 kg/m^2^ and 19.0% indicated they were following a specific diet for medical reasons. Based on the SCID, 58 (59.8%) met criteria for bipolar disorder and 39 (40.2%) for depressive disorder. More than 85% of the participants were taking psychiatric medications.

### Comparison of prevalence estimates of food insecurity

As hypothesized, the sample of adults with mood disorders had a higher proportion of individuals (36%) experiencing food insecurity (*p* < 0.001) compared to the general population sample (BCNS) (Table 1). The proportion of individuals reporting low-income status was also higher in adults with mood disorders (*p* < 0.05).

**Table 1:** Indicators of low income and food insecurity compared to regional survey data

Characteristic	Low-income status		Worry about food access^a^		Compromised diet^b^	
	Study (*n* = 97)	BCNS (*n* = 1,823)	Study (*n* = 97)	BCNS (*n* = 1,823)	Study (*n* = 97)	BCNS (*n* = 1,823)
Prevalence estimates (%) with 95% confidence intervals for income and food insecurity						
Income and food insecurity	40.2 (30.4–50.7)*	25.2 (23.2–27.3)	36.1 (26.6 to 46.5)**	7.3 (6.1–8.6)	19.6 (12.2–28.9)**	3.4 (2.6–4.3)

* *p* < 0.05, ** *p* < 0.0001.

^a^Question: in the past 12 months did you worry that there would not be enough to eat because of lack of money?

^b^Question: in the past 12 months did you not have enough food to eat because of lack of money?

### Food insecurity and nutrient intakes

The respondents who reported food insecurity had lower median carbohydrate and vitamin C intakes (Table 2). The assessment of nutrient intakes based on the EARs indicated that those reporting food insecurity had higher proportions of inadequate protein, folate and zinc intakes (*p* < 0.05). The food-insecure group also had a higher proportion of participants with excess total fat intakes as measured by the AMDR (*p* < 0.05).

**Table 2:** Comparisons of nutrient intakes by food security status

Measurement	Nutrient intakes according to food insecurity status^a^ (median, IQR^b^)		Nutrient intakes compared to *Dietary Reference Intakes* ^c^ and by food insecurity status^a^	
	Food insecure (*n* = 36)	Food secure (*n* = 61)	Food insecure (*n* = 36)	Food secure (*n* = 61)
Energy and macronutrients			*%* *>* *AMDR* ^*d*^ *(95% CI)*	
Energy (kilocalories)	2,406 (1,907; 2,875)	2,541 (1,802; 3,182)	–	–
Protein (% of calories)	14.0 (10.1; 18.4)	13.7 (9.4; 20.1)	5.5 (0.7–18.7)	0
Carbohydrates (% of calories)	47.8 (41.3; 57.3)*	52.9 (48.5; 59.8)	0.2 (0.1–19.5)*	11.4 (4.7–22.2)
Fibre (g)	21.5 (13.7; 29.4)	24.3 (15.3; 30.2)	–	–
Fats (% of calories)	35.4 (29.2; 45.0)*	34.2 (25.9; 38.9)	55.6 (38.1–72.1)*	29.5 (18.5–42.6)
			*%* *<* *EAR* ^*e*^ *(95% CI)*	
Protein (g/kg)	–	–	19.4 (6.5–32.4)*	4.9 (0.5–10.3)
Vitamins				
Vitamin B_6_ or pyridoxine (mg)	1.5 (1.0; 2.0)	1.5 (1.2; 2.2)	30.6 (16.3–48.1)	21.3 (11.9–33.7)
Vitamin B_9_ or folate (dietary folate equivalents)^f^	74.2 (39.7; 204.4)	114.5 (55.0; 225.4)	83.3 (67.2–93.6)*	52.5 (39.3–65.4)
Vitamin B_12_ or cobalamin (μg)	3.3 (1.9; 4.6)	3.5 (1.9; 5.1)	27.8 (14.2–45.2)	26.2 (15.8–39.1)
Vitamin C (mg)	80.5 (51.8; 180.7)*	128.8 (70.2; 216.7)	27.8 (14.2–45.2)	21.3 (11.9–33.7)
Trace minerals				
Iron (mg)	14.4 (10.2; 18.3)	16.0 (12.0; 23.8)	13.9 (4.7–29.5)	6.6 (1.8–15.9)
Zinc (mg)	8.9 (5.8; 11.8)	8.8 (6.4; 12.8)	52.8 (35.5–69.6)*	31.1 (19.9–42.3)

* *p* < 0.05.

^a^Food-insecure or secure based on respondents who answered yes to either of the following questions: (1) in the past 12 months did you worry that there would not be enough to eat because of lack of money; or (2) in the past 12 months did you not have enough food to eat because of lack of; food-secure based on respondents who answered no to both questions.

^b^Interquartile range.

^c^Reference values that are quantitative estimates of nutrient intakes to be used for planning and assessing diets for healthy people.

^d^Adequate macronutrient distribution ranges.

^e^Estimated average requirement.

^f^Dietary folate equivalents (DFE): values that adjust for the differences in absorption of food folate and synthetic folic acid; 1 mcg of DFE = 0.6 mcg of folic acid from fortified food or as a supplement taken with a meal = 1 mcg food folate; 0.5 mcg of folic acid from a supplement taken on an empty stomach.

### Food insecurity and psychological functioning

Based on the bivariate analysis, some associations were found between food insecurity and psychological functioning when measured as continuous variables (Table 3). Ham-D and YMRS scores were significantly higher (*p* < 0.05) in participants with compromised diet (i.e., answered affirmatively to the food insecurity screening question “In the past 12 months did you not have enough food to eat because of lack of money”). In addition, higher depression scores were found in those reporting low income (*p* < 0.05).

**Table 3:** Indicators of low income and food insecurity by psychological measures in adults with mood disorders (*n* = 97)

Psychological measure	Median and interquartile range					
	Low-income status		Worry about food access^a^		Compromised diet^b^	
	Yes (*n* = 39)	No (*n* = 58)	Yes (*n* = 35)	No (*n* = 62)	Yes (*n* = 19)	No (*n* = 78)
Global Assessment of Functioning	60 (51; 70)	65 (55; 75)	10 (3; 16)	9 (4; 14)	3 (1; 5)	3 (1; 4)
Hamilton Depression Scale	60 (51; 65)*	65 (60; 75)	9 (4; 16)	9 (4; 14)	3 (2; 7)*	2 (1; 4)
Young Mania Rating Scale	58 (43; 68)	65 (55; 75)	10 (3; 19)	9 (4; 14)	4 (2; 7)*	2 (1; 4)

* *p* < 0.05.

^a^Question: in the past 12 months did you worry that there would not be enough to eat because of lack of money?

^b^Question: in the past 12 months did you not have enough food to eat because of lack of money?

Crude prevalence ratios (PRs) of food insecurity and the psychological measures (dichotomized based on their defined cut-off scores) showed significant association between food insecurity and mania symptoms (Table 4). When Poisson regression was applied, the ratio of the proportion of respondents with high YMRS scores over the proportion with food insecurity was 2.37 (95% CI 1.49–3.75, *p* < 0.001). Low income was also consistently associated with worse scores on all psychological measures (adjusted PRs ranged from 2.68 to 2.88, *p* < 0.05). The variables in the final three Poisson regression models included the psychological measures (i.e., GAF, YMRS, Ham-D, respectively) age, sex, and income. The goodness-of-fit Chi-squared test results were not significant for all models (goodness-of-fit 51.34 to 55.01, *p*’s > χ^2^ (92) = 1.00) suggesting that the Poisson model form fit the data.

**Table 4:** Crude and adjusted prevalence ratios of food insecurity^a^, psychological, and income measures

Psychological measure	Crude prevalence ratio	Adjusted prevalence ratio^b^ (95% confidence interval, *p* value)	
		Food insecurity	Low income
GAF (<60)	1.49 (0.97–2.31, *p* = 0.072)	1.42 (0.88–2.28, *p* = 0.148)	2.68 (1.53–4.71, *p* = 0.001)
YMRS (>12)	4.72 (1.36–16.24, *p* = 0.006)	2.37 (1.49–3.75, *p* = 0.000)	2.71 (1.59–4.63, *p* = 0.000)
Ham-D (>7)	1.02 (0.71–1.48, *p* = 0.880)	0.94 (0.59–1.49, *p* = 0.783)	2.88 (1.64–5.06, *p* = 0.000)

^a^Answered yes to question 1 or 2: Question 1: In the past 12 months did you worry that there would not be enough to eat because of lack of money?; and Question 2: In the past 12 months did you not have enough food to eat because of lack of money?

^b^Prevalence ratios from final models that included food insecurity, age, sex, and income; income is included in table as the adjusted prevalence ratios were significant.

## Discussion

The results of this study indicated that food insecurity was more prevalent in adults with mood disorders when compared to a general population. Sample respondents who reported food insecurity had significantly lower intakes of carbohydrates and vitamin C. A higher proportion of the adults with mood disorders had potentially inadequate intakes of protein, folate, and zinc. Finally, food insecurity was consistently associated with worsened mania symptoms in adults with mood disorders.

This is the first study to report the relationships between food insecurity and selected nutrients in adults with mood disorders. As shown in other studies of food insecurity in different populations [14, 26–28], there is higher risk of inadequate intakes of essential nutrients which can have profound effects on mental health. Research conducted in this sample of adults with mood disorders has shown positive correlations between nutrient intakes and psychological function [29].

There are multiple interconnected ways in which food insecurity could contribute to poorer nutritional and psychological health, including through nutrient deficiencies and/or the body’s physiological response to the immense stress from not having the resources to feed oneself and/or one’s family [30–32]. When people experience food insecurity, they may be forced to consume food in socially unacceptable ways [33] which may affect psychological function directly or indirectly by altering nutrient intakes and metabolism. For example, reduced blood folate levels (that may be a result of compromised dietary folate intake due to food insecurity) may impair one-carbon metabolism, which has been implicated in the pathogenesis of psychiatric symptoms [34]. While previous studies have shown links between food insecurity and psychological symptoms such as depression, anxiety, and suicide ideation [6, 7, 28, 35, 36], our results are the first to show association with mania symptoms. One mechanism underlying this relationship may be due to suboptimal blood levels of vitamin C caused by either lower intakes and/or increased breakdown due to stress. With lower blood levels of vitamin C, disequilibrium between ascorbic acid and vanadium metabolism may occur and result in mania symptoms [37].

The consistent association between low-income status and worse scores on the psychological measures is not surprising as other studies have shown links with depression and low income [38]. However, to our knowledge, this study is the first to report associations with YMRS and GAF scores. While food insecurity is an independent determinant of health [39], cross-sectional surveys have also shown it is strongly linked with another health determinant, inadequate income [16, 40–42]. Unfortunately, the cross-sectional nature of these investigations and our study cannot determine the temporality of the relationships among food insecurity, low income, and mental health. However, longitudinal studies have shown that associations between food insecurity and depression are bidirectional [30, 43].

For several reasons, the results of this study should be interpreted with caution. Limitations include the modest sample size and that the food insecurity screening questions may not discriminate varying degrees of food access. However, previous studies indicate that short form measurement tools are appropriate to determine the subjective experiences of food insufficiency [44]. Measurement error between food insecurity and psychological functioning may be present as the two may be correlated (e.g., people in depressed mood states might report increased food insecurity regardless of their true status) and thereby yield a spuriously stronger positive association. However, the multivariate analysis did not show an association between food insecurity and Ham-D scores. Finally, most of the sample was taking psychiatric medications which may impact on food-related behaviours (e.g., increase appetite) [45] and therefore, increase food insecurity.

## Conclusions

Food insecurity is an important determinant of health and significant public health and social issue [39, 40]. This study extends the current body of food insecurity knowledge by providing prevalence estimates and reported associations with poorer nutrient intakes and psychological functioning in a sample of adults with mood disorders. While program and policy developments that strengthen food security would reduce health and social inequities [41, 42], it may also be speculated that such interventions may enhance the nutritional and psychological well-being of those with mood disorders. Future investigations should focus on interventions aimed at improving food security to establish its role in enhancing mental health.

## Abbreviations

AMDR: Adequate Macronutrient Distribution Ranges
BCNS: British Columbia Nutrition Survey
DSM-IV: Diagnostic and Statistical Manual of Mental Disorders, Fourth Edition
EAR: Estimated Average Requirement
Ham-D: Hamilton Depression scale
YMRS: Young Mania Rating Scale
GAF: Global Assessment of Functioning scale
